# Lessons learned: the first consecutive 1000 patients of the CCCMunich^LMU^ Molecular Tumor Board

**DOI:** 10.1007/s00432-022-04165-0

**Published:** 2022-07-07

**Authors:** Kathrin Heinrich, Lisa Miller-Phillips, Frank Ziemann, Korbinian Hasselmann, Katharina Rühlmann, Madeleine Flach, Dorottya Biro, Michael von Bergwelt-Baildon, Julian Holch, Tobias Herold, Louisa von Baumgarten, Philipp A. Greif, Irmela Jeremias, Rachel Wuerstlein, Jozefina Casuscelli, Christine Spitzweg, Max Seidensticker, Bernhard Renz, Stefanie Corradini, Philipp Baumeister, Elisabetta Goni, Amanda Tufman, Andreas Jung, Jörg Kumbrink, Thomas Kirchner, Frederick Klauschen, Klaus H. Metzeler, Volker Heinemann, C. Benedikt Westphalen

**Affiliations:** 1 Department of Medicine III and Comprehensive Cancer Center (CCC Munich LMU), University Hospital, LMU Munich, Marchioninistraße 15, 81377 Munich, Germany; 2 German Cancer Consortium (DKTK), Partner Site Munich, Munich, Germany; 3 Comprehensive Cancer Center (CCC Munich LMU), LMU University Hospital Munich, Munich, Germany; 4 Department of Neurology and Comprehensive Cancer Center (CCC Munich LMU), Ludwig Maximilians University, Munich, Germany; 5 Department of Apoptosis in Hematopoietic Stem Cells, Helmholtz Center Munich, German Center for Environmental Health (HMGU), Munich, Germany; 6 Department of Pediatrics, Dr Von Hauner Children’s Hospital, LMU, Munich, Germany; 7 Department of Obstetrics and Gynecology and Comprehensive Cancer Center (CCC Munich LMU), University Hospital, LMU Munich, Munich, Germany; 8 Department of Urology and Comprehensive Cancer Center (CCC Munich LMU), University Hospital, LMU Munich, Munich, Germany; 9 Department of Medicine IV and Comprehensive Cancer Center (CCC Munich LMU), University Hospital, LMU Munich, Munich, Germany; 10 Department of Radiology and Comprehensive Cancer Center (CCC Munich LMU), University Hospital, LMU Munich, Munich, Germany; 11 Department of General, Visceral und Transplantation Surgery and Comprehensive Cancer Center (CCC Munich LMU), University Hospital, LMU Munich, Munich, Germany; 12 Department of Radiation Oncology and Comprehensive Cancer Center (CCC Munich LMU), University Hospital, LMU Munich, Munich, Germany; 13 Department of Otorhinolaryngology, Head and Neck Surgery and Comprehensive Cancer Center (CCC Munich LMU), University Hospital, LMU Munich, Munich, Germany; 14 Department of Medicine II and Comprehensive Cancer Center (CCC Munich LMU), University Hospital, LMU Munich, Munich, Germany; 15 Department of Medicine V and Comprehensive Cancer Center (CCC Munich LMU), University Hospital, LMU Munich, Munich, Germany; 16 Institute of Pathology, Ludwig Maximilians University (LMU), Munich, Germany; 17 Department of Hematology, Cell Therapy and Hemostaseology, University Hospital Leipzig, Leipzig, Germany

**Keywords:** Precision oncology, Personalized medicine, Molecular tumor board, Targeted therapy, Comprehensive genomic profiling

## Abstract

**Purpose:**

In 2016, the University of Munich Molecular Tumor Board (MTB) was implemented to initiate a precision oncology program. This review of cases was conducted to assess clinical implications and functionality of the program, to identify current limitations and to inform future directions of these efforts.

**Methods:**

Charts, molecular profiles, and tumor board decisions of the first 1000 consecutive cases (01/2016–03/2020) were reviewed. Descriptive statistics were applied to describe relevant findings.

**Results:**

Of the first 1000 patients presented to the MTB; 914 patients received comprehensive genomic profiling. Median age of patients was 56 years and 58% were female. The most prevalent diagnoses were breast (16%) and colorectal cancer (10%). Different types of targeted or genome-wide sequencing assays were used; most of them offered by the local department of pathology. Testing was technically successful in 88%. In 41% of cases, a genomic alteration triggered a therapeutic recommendation. The fraction of patients receiving a tumor board recommendation differed significantly between malignancies ranging from over 50% in breast or biliary tract to less than 30% in pancreatic cancers. Based on a retrospective chart review, 17% of patients with an MTB recommendation received appropriate treatment.

**Conclusion:**

Based on these retrospective analyses, patients with certain malignancies (breast and biliary tract cancer) tend to be more likely to have actionable variants. The low rate of therapeutic implementation (17% of patients receiving a tumor board recommendation) underscores the importance of meticulous follow-up for these patients and ensuring broad access to innovative therapies for patients receiving molecular tumor profiling.

**Supplementary Information:**

The online version contains supplementary material available at 10.1007/s00432-022-04165-0.

## Introduction

In some malignancies such as colorectal cancer (Van Cutsem et al. 2016), melanoma (Sosman et al. 2012) and breast cancer (Gennari et al. 2021), focused biomarker testing is standard of care. In other malignancies, such as non-small-cell-lung-cancer (Shaw et al. 2013) and AML (Heuser et al. 2020), multi-gene next-generation sequencing (NGS) has been established in clinical routine (Mosele et al. 2020). Aside from biomarkers used in clinical practice, various new therapeutic targets are currently investigated in molecularly guided clinical trials.

In 2017, Pembrolizumab was the first drug to receive tissue/site-agnostic approval for microsatellite instability-high (MSI-H) or mismatch repair-deficient (dMMR) solid tumors (FDA 2017). In 2020, this label was extended for metastatic solid tumors with high tumor mutational burden (TMB-H; ≥ 10 mutations/megabase). In 2018 Larotrectinib, a highly selective TRK inhibitor was the second drug to be granted FDA-approval, regardless of histology, for solid tumors harbouring NTRK gene fusions after durable antitumor activity was shown across three different trials (Drilon et al. 2018). Entrectinib, another TRK inhibitor, has received histology-agnostic approval for NTRK-positive solid tumors for adults and pediatric patients 12 years of age (FDA 2019). With Selpercatinib and Pralsetinib, two RET-inhibitors are now available for patients with RET-altered thyroid or lung cancer (Drilon et al. 2020; Wirth et al. 2020; Subbiah et al. 2021). Pemigatinib received accelerated approval in patients with advanced or metastatic cholangiocarcinoma harbouring FGFR2 rearrangements or fusions and is currently being investigated in a phase III trial in first-line treatment of metastatic cholangiocarcinoma (Abou-Alfa et al. 2020, Bekaii-Saab et al. 2020). In 2021, the FDA granted accelerated approval to Sotorasib, the first KRAS-inhibitor for patients with KRAS p.G12C-mutated lung cancer (Skoulidis et al. 2021). This list—without aiming to be complete—illustrates the growing impact of targeted therapy in clinical practice.

The use of comprehensive genomic profiling (CGP) has affected diagnostic and therapeutic management in many advanced malignancies (Hyman et al. 2017). While tumor multi-gene NGS testing may have been the exception a few years ago, routine application is now recommended by ESMO for certain tumor entities such as NSCLC, cholangiocarcinoma, prostate, or ovarian cancer (Mosele et al. 2020). Testing for TMB is recommended for patients with well- and moderately differentiated neuroendocrine tumors (NETs), cervical, salivary gland, thyroid and vulvar cancers (Mosele et al. 2020).

Precision oncology, as a patient-centric approach, aims to find “the right drug for the right patient at the right time” (Subbiah and Kurzrock 2018). To meet this goal, it is necessary to consider not only the genomic profile of the tumor but also the clinical situation of the patient as well as the patient’s perspective and expectations (Subbiah and Kurzrock 2018). Decreasing cost, improved turnaround time and increased availability have led to a broader usage of CGP in clinical practice. With increased usage of CGP, more alterations of unclear clinical significance are found (Moscow et al. 2018).

One way to embed precision oncology in clinical routine is the implementation of dedicated precision oncology programs and/or molecular tumor boards (MTB). An MTB is an interdisciplinary meeting during which experts on precision oncology from different specialties discuss the results of genomic testing. Based on this meeting, a therapeutic recommendation is given. By now, there are several reports about single-center experiences with MTBs (Schwaederle et al. 2014, Tafe et al. 2015, Brock and Huang 2017, Burkard et al. 2017, Bernhardt et al. 2020, Kato et al. 2020). However, therapy recommendations made at those tumor boards show great heterogeneity (Rieke et al. 2018). This is mostly due to heterogenous levels of evidence and different opinions regarding the therapeutic value of potential therapeutic targets. Several different classification systems to rank the potential clinical value of a found alteration have already been proposed and compared (Leichsenring et al. 2019).

Despite the promising results of several basket trials leading to tumor-agnostic FDA-approval (8, 11, 12, 14, 15, and 27–29), questions remain as to what extent these results provide an impact to real world cancer patients. Two meta-analyses of phase 1 and phase 2 trials showed improved response as well as improved survival data for patients treated with biomarker-based treatment strategies (Schwaederle et al. 2015, 2016). For broader use of next-generation sequencing (NGS), the results are far more sobering. Only a small percentage of patients that undergo molecular testing receive targeted therapy in the end (Massard et al. 2017; Tredan et al. 2019). There has been evidence that patients do not seem to benefit from targeted therapy when examining response rate, progression free survival or overall survival (Le Tourneau et al. 2015; Massard et al. 2017; Tredan et al. 2019). Due to the uncertainties surrounding the value of CGP in routine clinical practice, precision medicine programs should continuously document and analyze the clinical characteristics and outcomes of patients discussed in their MTB.

In the following analysis, the results and experiences of the precision oncology program at the University Hospital Munich will be presented and discussed.

## Material and methods

### Molecular Tumor Board

At the University Hospital Munich, a biweekly interdisciplinary MTB was established as part of the Precision Oncology Program in 2016. Due to a rising case load, the MTB started to meet on a weekly basis in early 2020. With the advent of the COVID-19 pandemic, the MTB was switched to a virtual meeting. The virtual format was maintained to allow access to the MTB for the growing number of external partners.

In this tumor board, clinicians, pathologists, tumor geneticists and experts for precision oncology discuss the results of CGP within a patient’s clinical context. Recommendations are graded according to the ESMO Scale for Clinical Actionability of molecular Targets (ESCAT) and National Center for Tumor Diseases (NCT) levels of evidence (Mateo et al. 2018) to ensure reproducible interpretation of results. To support the evaluation and interpretation of CGP results, an on-site literature database was created. The clinical implementation of the recommendation remains the responsibility of the primary care team. While some MTBs at other centers discuss cases before testing to decide on the necessity of CGP, the MTB in Munich generally discusses cases only after the results are available (see below).

### Workflow

Extended molecular testing is initiated by the organ-/entity-specific tumor board or after consultation with the coordinator of the precision oncology program. Several patient characteristics can help identify patients that might benefit from CGP:

- Patients suffering from advanced disease with no further “standard of care” therapeutic options.
- Patients presenting with an unusual clinical presentation or disease course for the respective disease or suffering from a rare entity or a rare pathological subtype.
- Patients should qualify for experimental treatment concerning clinical condition and life expectancy.

Testing is conducted in the local department of pathology (Department of Pathology of the LMU). Online registration with the MTB team happens simultaneously to ensure that the case will be discussed in the tumor board as soon as the results are available. Cases are submitted to the MTB via an online registration system based on the Clinical Workplace Program of the hospital.

### Diagnostics

#### Sequencing assays

At the CCC^LMU^ different types of extended molecular diagnostic tests have been used, most of them available through the local pathology department. In some cases, testing was performed by commercial providers. In-house NGS included several targeted gene panels, a 52-gene panel (Oncomine™ Focus Assay, ThermoFisher Scientific) which was replaced by a 161-gene panel in 2019 (Oncomine™ Comprehensive Assay v3 (OCAv3), ThermoFisher Scientific), which also included BRCA testing. Both panels allowed analysis of DNA and RNA to simultaneously detect single nucleotide variants (SNVs) and insertions/deletions (indels) as well as copy number variations (CNVs) and gene fusions. Moreover, in 2019, the Oncomine Tumor Mutational Load Assay (ThermoFisher Scientific) was added to the molecular pathology repertoire. In-house testing can be performed on tumor tissue (FFPE) or on liquid biopsies. Liquid biopsies can be obtained from blood, from cerebral spinal fluid or other body fluids such as ascites.

Young patients with rare cancers can be referred to the NCT Master program (Horak et al. 2017) to receive whole exome and transcriptome sequencing. Those patients are discussed in the MTB of the NCT Master program. To participate in the NCT Master program, a new biopsy must be performed to obtain a fresh frozen sample.

Patients can be referred to the MTB from external hospitals or physicians with already performed CGP to discuss the results.

### Follow-up

Retrospective follow-up was conducted by review of electronical and paper charts of the first 1000 patients. Baseline characteristics were taken from the most recent physician’s report. Results of molecular testing and therapy recommendation were taken from the decision of the Molecular Tumor Board.

## Results

Of the first 1000 patients prospectively enrolled in the program, 914 (91.4%) received genomic testing. Six patients were presented to the program to discuss the indication for extended molecular testing. The remaining 80 patients did not undergo molecular diagnostics due to clinical deterioration, death, or lack of clinical indication for molecular testing after internal discussions with the primary care team.

Across the study period, the Precision Oncology Program experienced a rapid growth of case numbers: in 2016, 21 patients were studied by NGS, in 2017, 134 patients, in 2018, 260 patients and in 2019, 448 patients. In 2020, 609 patients had undergone testing. We limited the present analysis to the first 1000 patients overall. 51 of them were diagnosed in 2020 (last patient in: 09.03.2020).

### Patient characteristics

Of the 914 tested patients, 383 (42.0%) were male and 534 (58.0%) were female. The slight imbalance in patient sex is due to the large gynecological oncology program at University of Munich. In 2017, more than 60% of patients were female. This number dropped to 53% in 2020, demonstrating the expansion of the program. In the non-gynecological cohort, the ratio between male and female patients was 54.4% and 45.6%, respectively. Most patients (903; 98.8%) suffered from solid malignancies that were metastasized in 748 (82.8%) cases at the time of CGP. Median age of all patients was 56 years (range 14–86, see Table 1).

**Table 1:** Baseline characteristics of the first 1000 patients

Baseline characteristics (*n* = 1000)	
Age	Median: 56 years; range 14–86 years
Gender	383 (42.0%) male, 534 (58.0%) female
Diagnosis (selected); *n* (%)	
Breast cancer	148 (16.2)
Biliary tract cancer	56 (6.1)
Cervical cancer	15 (1.6)
Colorectal cancer	100 (10.9)
Cancer of unknown primary	42 (4.6)
Gastric cancer	14 (1.5)
Glioblastoma	15 (1.6)
Head and neck cancer	11 (1.2)
Melanoma	15 (1.6)
Neuroendocrine malignancies	23 (2.5)
NSCLC	61 (6.7)
Ovarian cancer	37 (4.0)
Pancreatic cancer	71 (7.8)
Prostate cancer	10 (1.1)
Sarcoma	54 (5.9)
Thyroid cancer	46 (5.0)
Urinary tract cancer	24 (2.7)

In total, patients from 19 different clinical departments (excluding external referrals) were presented to the MTB. Most patients suffered from gastrointestinal (30.9%) or gynecological malignancies (23%). Around 10% of cases, respectively, were diagnosed with endocrine tumors or lung cancer. Roughly 5% of patients, respectively, presented with cerebral malignancies, CUP syndrome, sarcoma, or urogenital tumors. Of the 914 patients, 40.6% were diagnosed with rare cancers (incidence of < 6/100.000) (ESMO 2022), which is in contrast to the general incidence of rare cancers (20–25% of all cancers).

In most cases (*n* = 821; 89.8%), tumor tissue was used to perform CGP. In 38%, tissue from the primary tumor was used for CGP. In 54.7%, metastases were analyzed. In 77 cases (8.4%), liquid biopsies taken from blood, cerebral spinal fluid or ascites were used. 85% of CGP was performed at the local pathology department. 11.6% were either discussed based on CGP or tested externally. 1.4% of the patients were referred to the NCT MASTER program. On average, patients underwent CGP 36 months after they were first diagnosed. Median time from initial diagnosis to extended molecular testing was 18 months (range 0–490 months) and differed between primary diagnosis of the patients (see Supplementary Table S1).

### Outcome

**Fig. 1:**
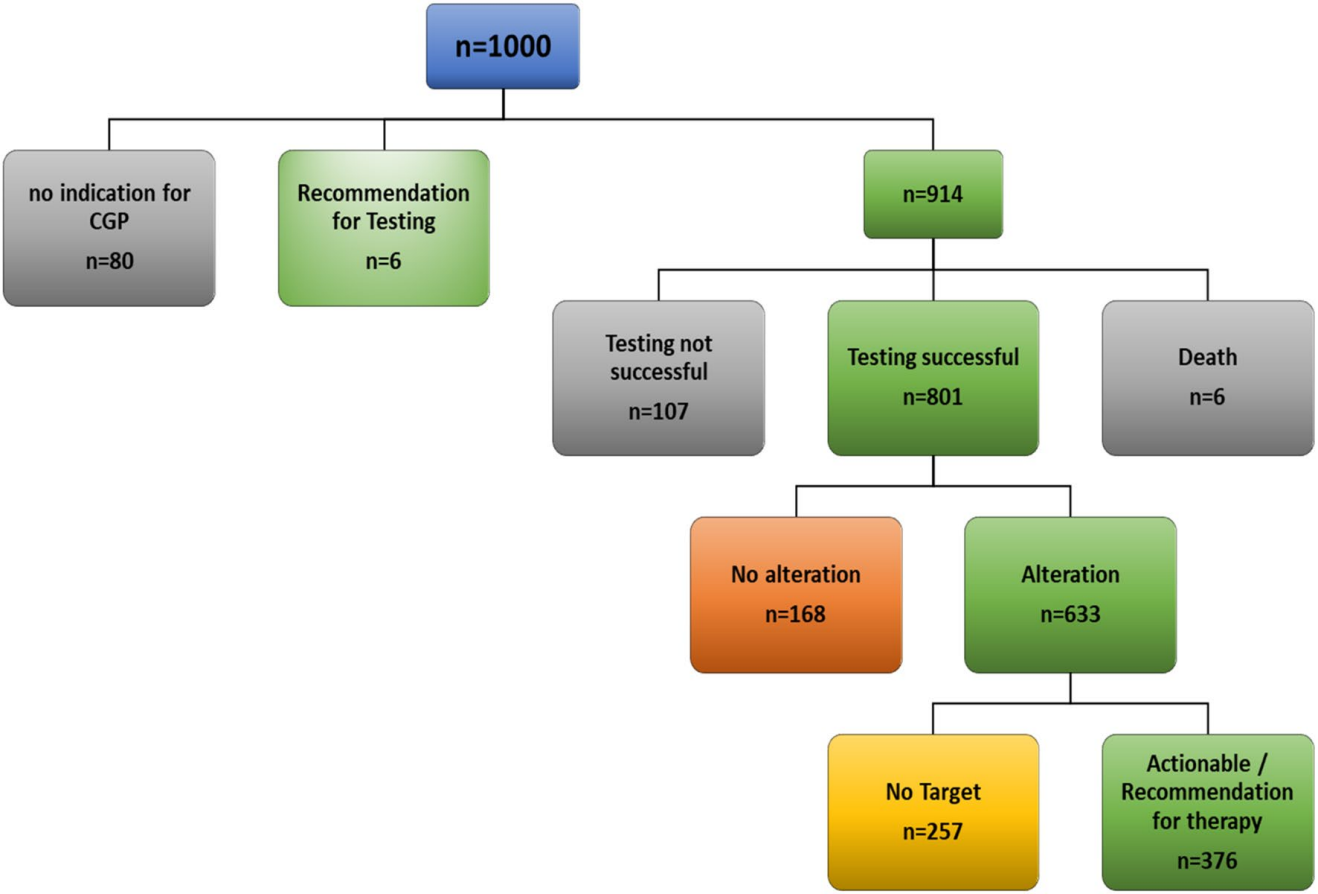
Consort diagram of the first 1000 patients

Of the 914 patients that underwent CGP, 6 died before the analysis was finalized or their case could be presented in the MTB. In 107 cases (11.7%), CGP was not successful due to technical reasons. Main reasons for technical failure of analysis were insufficient material or insufficient quality of the sample used. In 41.1% of patients (*n* = 376), a therapeutic recommendation was given. An alteration that could not be therapeutically addressed was found in 28.1% (*n* = 257). A total of 18.4% (*n* = 168) presented with no alteration in the genes tested (Fig. 1 and Table 2).

**Table 2:** Outcome in regard to the respective panel

Panel	Total number	Actionable/Recommendation for therapy	Alteration, not druggable	No alteration	Testing not successful
In-house	337	122 (36.2%)	57 (16.9%)	99 (29.4%)	59 (17.5%)
In-house (after application of the comprehensive (OCAv3) and TMB panel)	434	173 (40.0%)	163 (37.6%)	61 (14.1%)	37 (8.5%)
Commercial	94	51 (54.3%)	32 (34.0%)	2 (2.1%)	9 (9.6%)

In the subset of patients (*n* = 45) that were diagnosed from liquid biopsies, testing was technically successful in roughly 90%, and 35% of those patients received a recommendation from the tumor board, thus demonstrating the feasibility of this approach in this exploratory subgroup.

### Therapeutic recommendations

There are different types of recommendations the MTB could offer (see Table 3). Most of the recommendations included the use of targeted therapies such as tyrosine kinase inhibitors (25.3%) or mTOR-Inhibitors (19.7%). In 10.6%, use of immune checkpoint inhibitors was recommended. Further diagnostic steps or a referral for genetic counseling were recommended in 10% of patients. Other possible recommendations were the inclusion in a clinical trial (8.5%) or a change in treatment management (0.8%) if CGP led to a change in diagnosis. In some cases, additional diagnostics (3.8%) or genetic counseling (7.2%) was recommended. Sometimes, recommendations overlapped, especially when off-label use or participation in clinical trials was an option. A significant number of cancers showed alterations that were not actionable at the time of MTB discussion. Among the found alterations that led to no therapeutic recommendation, the most frequent were TMB low (61.1%), KRAS (37.8%) and TP53 (33.5%) (see Table 4). In most cancers, more than one pathogenic alteration was found. The recommendation rate In between 2017 and 2019, the percentage of therapeutic recommendations remained relatively stable (40.2%). For the first 51 patients in 2020, the rate of recommendations rose to 45.1% and has remained relatively stable in that range until today (data not shown).

**Table 3:** Therapeutic recommendations of the first 1000 patients

Therapeutic recommendation	*n*	Frequency (%)
TKI	95	25.3
mTOR inhibitor	74	19.7
Immune-checkpoint-inhibitor	40	10.6
PARP inhibitor	34	9.0
Clinical trial	32	8.5
BRAF/MEK inhibition	27	7.2
Genetic counselling	27	7.2
Monoclonal antibody	23	6.1
IDH1/2 inhibitor	17	4.5
Additional diagnostics	14	3.8
Alpelisib	10	2.7
Trametinib/hydroxychloroquine	10	2.7
Androgen-receptor blockade	9	2.4
CDK4/6 inhibitor	9	2.4
Change in management	3	0.8

*TKI* tyrosine kinase inhibitor, *mTOR* mechanistic target of rapamycin, *PARP* poly-[ADP-ribose-]polymerase, *IDH* isocitrat-dehydrogenase; *CDK* cyclin-dependent kinase

**Table 4:** Not actionable alterations

Alteration	*n*	Frequency (%)
TMB LOW	157	61.1
KRAS	97	37.8
TP53	86	33.5
PIK3CA	17	6.6
MYC	14	5.4
ARID1A/2	10	2.8
CDKN2A	10	2.8
NRAS	9	3.5
SMAD 4	8	3.1
APC	7	2.7
CTNNB1	5	2.9
BRAF (non V600E)	3	1.2
PTEN	3	1.2

### Tumor mutational burden

In 233 patients, analysis of tumor mutational burden (TMB) was performed. Results are presented in Table 5.

**Table 5:** Results of analysis of tumor mutational burden in 233 patients

TMB	*N*	%
Low* (≤ 5 mutations/Mb)	157	67.38
Intermediate* (> 5/ < 20 mutations/Mb)	65	27.90
High* (≥ 20/ < 50 mutations/Mb)	9	3.86
Very high* (≥ 50 mutations/Mb)	2	0.86
< 10 Mutations/Mb	208	89.27
≥ 10 Mutations/Mb	25	10.73

*Definition according to Riviere et al. (2020)

### Outcome of CGP depending on primary diagnosis

**Fig. 2:**
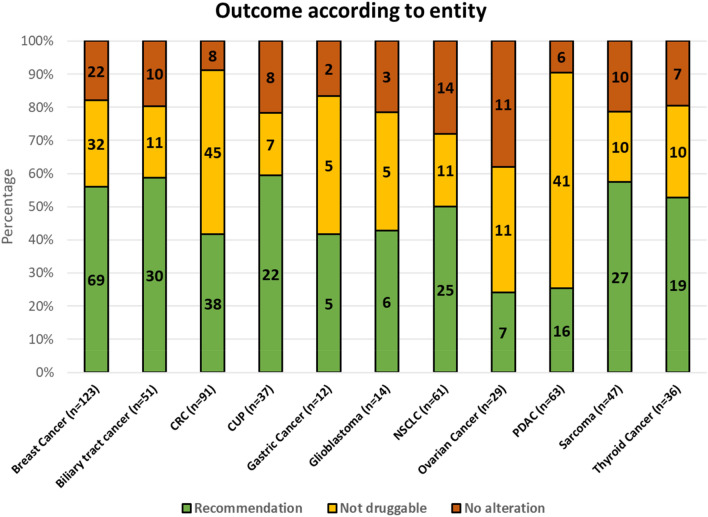
Outcome of NGS regarding diagnosis; alphabetical order; Absolute numbers for respective category in bars

Differences between entities were observed when looking at the frequency of therapy recommendations (as summarized in Fig. 2). In patients with biliary tract cancer, molecular profiling led to a therapeutic recommendation in almost 60% of cases. In patients with pancreatic cancer, an alteration was found in around 90% (mostly Kras mutations) but in only 28% did this alteration lead to a therapeutic recommendation. As testing success was comparable in between groups (83% in breast cancer vs 92% in PDAC) and the frequency of any alteration was similar between breast and pancreatic cancer (85% vs 92%), these differences are based on the molecular and therapeutic landscape of each disease.

### Follow-up

Retrospective follow-up showed that only around 19% of therapeutic recommendations were put into practice. In 63%, a realization of the recommendation was planned or the process of implementing the recommendation was unknown. Those 63% comprise patients that were presented to the MTB in earlier treatment lines and who were still under active tumor therapy. Furthermore, in some cases, approval of off-label use by the health insurance company was pending. Patients, where implementation of recommendation is unclear, are mostly patients from external partners who are lost to follow-up.

## Discussion

Here, we report data of the first 1000 patients of our Precision Oncology Program between 2016 and 03/2020. One of the main and most sobering findings is the low rate of realized treatment recommendations. 41.4% of tested patients received a therapeutic recommendation but only 17% of those recommendations were put into practice. These rates are in line with previous published retrospective results by Tannock et al., stating that “for or every 1000 patients […] about 400 will have a targetable mutation, about 120 will receive a matched drug” (Tannock and Hickman 2019). There are various reasons that contribute to this result within our program. One reason is that, especially in the beginning of the program, patients were often presented to the MTB at a very advanced stage so that they did not receive experimental treatment due to rapid clinical deterioration. Furthermore, access to targeted treatments proved difficult in the beginning of the program for both off-label treatments and clinical trials.

The fact that inclusion in a clinical trial was recommended in only 8% shows that a closer cooperation with an early clinical trial program is imperative to increase the potential benefit for patients undergoing CGP (Dienstmann et al. 2020). Six years into the program, we feel that we have the obligation to share our early experiences with the scientific community to underscore the need to push for a more integrative precision oncology approach where diagnostic and therapeutic interventions, access to treatment, and meticulous follow-up are tightly interwoven. We see that in smaller subgroups, such as gynecological cancers, a dedicated, structured follow-up, including survival and efficacy endpoints, is feasible within our program (Sultova et al. 2021a, b). Other programs have shown that structured follow-ups regarding outcomes are possible in the setting of clinical trials (Horak et al. 2021) as well as in structured programs (Bitzer et al. 2020). Based on our experiences, we have implemented comprehensive measures to improve the shortcomings demonstrated in the early days of our program. A clinical registry was started to evaluate efficacy and survival in patients enrolled in the MTB. All patients undergoing CGP should be included in this registry to ensure a structured evaluation and collection of clinical data, especially regarding response and survival.

This professionalization and process optimization was accompanied by a rapid growth in case numbers, showing the need and broad usage of CGP in clinical routine at a large academic center. Due to the increase of case numbers, the MTB changed from being held every other week to weekly board discussions. The expansion of the program was mainly driven by the implementation of a network of internal and external partners. A key characteristic of the CCC Munich^LMU^ MTB is the interdisciplinary nature of the platform. Patients were referred from 19 different clinical departments (excluding external referrals) and experts from more than 10 medical specialties regularly attend the meeting and contribute to shaping the program. This has resulted in several scientific collaborations that underscore the potential of a dedicated Precision Oncology Program to serve as an interdisciplinary platform to foster academic innovation (Rohrmoser et al. 2020; von Baumgarten et al. 2020; Rodler et al. 2021; Sultova et al. 2021a, b). To facilitate cooperation with external partners, plans for a virtual MTB were made as early as 2018.

With the advent of the COVID-19 pandemic, the MTB was changed to a virtual format. Importantly, changing the format of the MTB did not impact growth of the program. On the contrary, while in the 12 months before the first lockdown in Germany, only 493 patients were presented to the program, in the 12 months following the lockdown, numbers increased to 617 patients. This growth underscores the clinical need for the platform. Owing to the virtual format, onboarding of external partners became more feasible and referrals increased significantly. To sustain the growth of the program and foster trans-sectoral collaboration, the MTB will remain in its virtual format.

Previously published analyses suggested that personalized treatment leads to improved outcomes and fewer toxic deaths (Schwaederle et al. 2015). Nevertheless, the reality of the situation seems to be that only a small number of patients screened in precision oncology trials receive targeted agents, and only a minority of treated patients benefit from treatment (Moscow et al. 2018). This is in line with our experiences since clinical utility of CGP seems to depend heavily on the primary diagnosis of a given patient and the availability of viable therapeutic options. While 59% of patients presenting with biliary tract cancer received a recommendation by the tumor board, patients suffering from PDAC received a recommendation in 25% of cases. This is in line with subgroup analysis from the MOSCATO01 trial (Verlingue et al. 2017) and another important aspect to consider for future patients of the program.

## Conclusion

The present analysis shows that a Precision Oncology Program with an interdisciplinary MTB is feasible at a large university hospital. There are several limitations to this analysis. First, this is a single-center experience. Second, the decision to include patients into the program was up to the treating physician, which might have led to a selection bias not only regarding primary diagnosis but also regarding the timing of testing. Third, due to the interdisciplinary management of patients from various department, follow-up has been challenging during the implementation phase of the program but has already been conducted within smaller subgroups (Sultova et al. 2021a, b). To provide high-quality, real world evidence, a dedicated follow-up program, including a prospective and retrospective registry, has been implemented, which will allow us to further evaluate the benefit to patients of such a program.

Even though the sobering realization of only 17% of recommendations is in line with previously published results (Tannock and Hickman 2019), a careful selection of the right point in time, the right patient and the right diagnostic tool, as described by Subbiah and Kurzrock to improve outcomes, seems to be the most important lesson from the first 1000 cases (2018). The identification of patients who might benefit from GCP is crucial. So too is testing in earlier treatment lines to ensure that patients are in a condition of undergoing an experimental treatment when receiving a therapeutic recommendation (Subbiah and Kurzrock 2018). When cooperating with a department containing an early clinical trial unit, MTB can serve as a screening tool for early, biomarker-guided clinical trials (Dienstmann et al. 2020). As mentioned before, even though we lack structured follow-up and survival data, we feel responsible to share our experiences with the scientific community to promote an integrative approach to precision oncology.

## Supplementary Information

Below is the link to the electronic supplementary material.

Supplementary file1 (DOCX 13 kb)
