# Supplementary material for: Lessons learned: the first consecutive 1000 patients of the CCCMunichLMU Molecular Tumor Board
Source: J Cancer Res Clin Oncol. 2022 Jul 7;149(5):1905–15. doi: 10.1007/s00432-022-04165-0 (PMC9261163; doi:10.1007/s00432-022-04165-0)
Supplement: Supplementary file 1 — Supplementary file1 (DOCX 13 kb) [file 432_2022_4165_MOESM1_ESM.docx]

| ***DIAGNOSIS*** | **N** | **MEDIAN** | **RANGE** | **MISSING** |
| --- | --- | --- | --- | --- |
| *CNS* | 45 | 41.5 | 0 – 490 | 1 |
| *CUP* | 42 | 4.0 | 0 – 30 | 2 |
| *ENDO* | 87 | 20.0 | 0 – 389 | 0 |
| *GI* | 282 | 12.0 | 0 – 156 | 15 |
| *GYN* | 210 | 36.5 | 0 – 281 | 2 |
| *HEAD AND NECK* | 20 | 17.0 | 1 – 142 | 1 |
| *HEME* | 11 | 24.5 | 0 – 136 | 1 |
| *LUNG* | 76 | 10.0 | 0 – 237 | 4 |
| *SARCOMA* | 56 | 21.0 | 0 – 273 | 1 |
| *SKIN* | 26 | 40.0 | 0 – 284 | 0 |
| *URO* | 46 | 14.5 | 0 – 205 | 0 |

Table 6: Supplementary Table, Median time (in months) from first diagnosis of malignant disease to NGS
